# Lipid starvation and hypoxia synergistically activate *ICAM1* and multiple genes in an Sp1-dependent manner to promote the growth of ovarian cancer

**DOI:** 10.1186/s12943-015-0351-z

**Published:** 2015-04-08

**Authors:** Shiro Koizume, Shin Ito, Yoshiyasu Nakamura, Mitsuyo Yoshihara, Mitsuko Furuya, Roppei Yamada, Etsuko Miyagi, Fumiki Hirahara, Yasuo Takano, Yohei Miyagi

**Affiliations:** Molecular Pathology and Genetics Division, Kanagawa Cancer Center Research Institute, 2-3-2 Nakao, Asahi-ku, Yokohama, 241-8515 Japan; Departments of Pathology, Yokohama City University Graduate School of Medicine, 3-9 Fukuura, Kanazawa-ku, Yokohama, 236-0004 Japan; Obstetrics, Gynecology and Molecular Reproductive Science, Yokohama City University Graduate School of Medicine, 3-9 Fukuura, Kanazawa-ku, Yokohama, 236-0004 Japan

**Keywords:** Hypoxia, Sp1, Lipid starvation, ICAM1, Ovarian cancer

## Abstract

**Background:**

Elucidation of the molecular mechanisms by which cancer cells overcome hypoxia is potentially important for targeted therapy. Complexation of hypoxia-inducible factors (HIFs) with aryl hydrocarbon receptor nuclear translocators can enhance gene expression and initiate cellular responses to hypoxia. However, multiple molecular mechanisms may be required for cancer cells to adapt to diverse microenvironments. We previously demonstrated that a physical interaction between the ubiquitously expressed transcription factor Sp1 and HIF2 is a major cause of *FVII* gene activation in poor prognostic ovarian clear cell carcinoma (CCC) cells under hypoxia. Furthermore, it was found that *FVII* activation is synergistically enhanced when serum-starved cells are cultured under hypoxic conditions. In this study, we investigated whether HIFs and transcription factor Sp1 cooperate to activate multiple genes in CCC cells under conditions of serum starvation and hypoxia (SSH) and then contribute to malignant phenotypes.

**Methods:**

To identify genes activated under hypoxic conditions in an Sp1-dependent manner, we first performed cDNA microarray analyses. We further investigated the molecular mechanisms of synergistic gene activations including the associated serum factors by various experiments such as real-time RT-PCR, western blotting and chromatin immunoprecipitation. The study was further extended to animal experiments to investigate how it contributes to CCC progression *in vivo*.

**Results:**

*ICAM1* is one such gene dramatically induced by SSH and is highly induced by SSH and its synergistic activation involves both the mTOR and autonomously activated TNFα-NFκB axes. We identified long chain fatty acids (LCFA) as a major class of lipids that is associated with albumin, a serum factor responsible for synergistic gene activation under SSH. Furthermore, we found that ICAM1 can be induced *in vivo* to promote tumor growth.

**Conclusion:**

Sp1 and HIFs collaborate to activate genes required for the adaptation of CCC cells to severe microenvironments, such as LCFA starvation and hypoxia. This study highlights the importance of transcriptional regulation under lipid starvation and hypoxia in the promotion of CCC tumor growth.

**Electronic supplementary material:**

The online version of this article (doi:10.1186/s12943-015-0351-z) contains supplementary material, which is available to authorized users.

## Background

Cancer cells adapt to hypoxic microenvironments using aberrant vasculature to enable tumor growth. Hypoxia-inducible factors (HIFs) 1α and 2α are major transcription factors required for adaptive responses to hypoxia. HIFs in complex with the aryl hydrocarbon receptor nuclear translocator (ARNT) can bind hypoxia response elements (HREs) of target genes, where they act to enhance transcription [1]. This conventional mechanism regulates genes such as *VEGF* and *HO* to improve relatively mild hypoxic conditions [2,3]. However, many tumor types, especially those of pancreatic and cervical origin, are known to experience more severe hypoxic stresses [4].

To respond to dynamic changes in hypoxic conditions combined with variable nutrient, growth factor, and hormone environments, tumor cells employ multiple stress response mechanisms. In addition to HIF signaling, endoplasmic reticulum (ER) stress followed by the unfolded protein response (UPR) is another cellular response involved in adaptation to severe hypoxia [3]. The UPR can stimulate the transcription of additional genes required for tumor survival and growth.

Mammalian target of rapamycin (mTOR) signaling is also important for adaptation of cancer cells to hypoxia. mTOR is a serine/threonine kinase and a regulator of protein expression at the translational level [5]. mTOR is activated via phosphorylation of its serine residues, and its activation regulates levels of cellular nutrients, energy availability, and growth factors [6]. In general, mTOR becomes inactivated in hypoxic tumor tissues. However, it is known that mTOR dysregulation promotes the translation of HIF1 [5] and cooperates in responses to hypoxic conditions, suggesting that the interaction between the HIF- and mTOR-signaling pathways is involved in adaptation to hypoxia.

We previously demonstrated that the *FVII* gene, which encodes blood coagulation factor VII (fVII) [7], can be induced in ovarian clear cell carcinoma (CCC) cells in response to hypoxia [8,9]. Ectopic expression of this pro-coagulant promotes phenotypic changes in cancer cells [8,10]. We also demonstrated that a physical interaction between the ubiquitously expressed transcription factor Sp1 [11] and HIF2 is a major cause of *FVII* activation in CCC cells under hypoxic conditions [12]. Furthermore, *FVII* activation is synergistically enhanced via a UPR-independent pathway when serum-starved cells are cultured under hypoxic conditions, explaining why thrombosis frequently occurs in CCC patients [12]. However, little is known of the mechanisms underlying this synergism.

Ovarian cancer is most the malignant of the gynecological neoplasms [13,14]. CCC in particular is highly resistant to chemotherapy and has a poor prognosis [14]. We hypothesized that genes regulated by the above-described Sp1-dependent mechanism are responsible for adaptive responses to hypoxia with a severity and/or duration that is characteristic of CCC tissues. The aim of this study was to test this hypothesis. We first tested whether multiple genes are activated under hypoxia in an Sp1-dependent manner in CCC cells and whether such transcriptional activations occur synergistically during serum starvation. We further investigated the molecular mechanisms of synergistic gene activation, including regulation by associated serum factors. The study was further extended to investigate *ICAM1* gene expression *in vivo* and its contribution to CCC progression.

## Results

### Sp1 and HIFs cooperate to activate multiple genes under hypoxia

**Figure 1:**
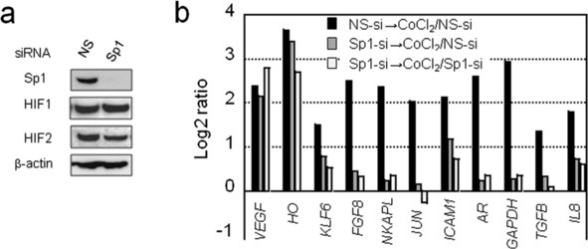
**cDNA microarray analysis of genes activated by CoCl**_**2**_ **stimulation in an Sp1-dependent manner. a)** western blot analysis of lysates from cells transfected with non-specific (NS) or Sp1 siRNA. **b)** log2 ratio of mRNA levels transfected with NS- or Sp1-siRNAs and then cultured for 24 h. Cells were further cultured for an additional 4 h under stimulation with CoCl_2_ (500 μM) or treatment with a vehicle control.

To identify genes activated under hypoxic conditions in an Sp1-dependent manner, we first performed cDNA microarray analyses. We used CoCl_2_ treatment under normoxia, as we found previously that *FVII* induction with this hypoxia-mimicking agent is higher than induction observed by maintaining cells in 1% O_2_ [12]. A CCC cell line (OVSAYO) was first transfected with Sp1-siRNA (Sp1-si) or negative control siRNA (NS-si) and then cultured with (→CoCl_2_) or without CoCl_2_. Western blotting showed that Sp1 knockdown did not affect HIF induction (Figure 1a). Basal promoter activity of *VEGF* [15] and *HO* [16] genes can be regulated by Sp1. However, the expression ratios of these HRE-dependent genes in CoCl_2_ treated cells vs. non-treated cells were largely unchanged between control cells (NS-si → CoCl_2_ treatment/NS-si) and Sp1-silenced cells (Sp1-si → CoCl_2_ treatment/NS-si and Sp1-si → CoCl_2_ treatment/Sp1-si) (Figure 1b). In contrast, expression of multiple genes in the presence of CoCl_2_ was decreased by Sp1 silencing (Figure 1b and Additional file 1: Table S1). Among them, the *GAPDH* and *TGFB* genes have also been reported to be HRE-dependent [17,18].

**Figure 2:**
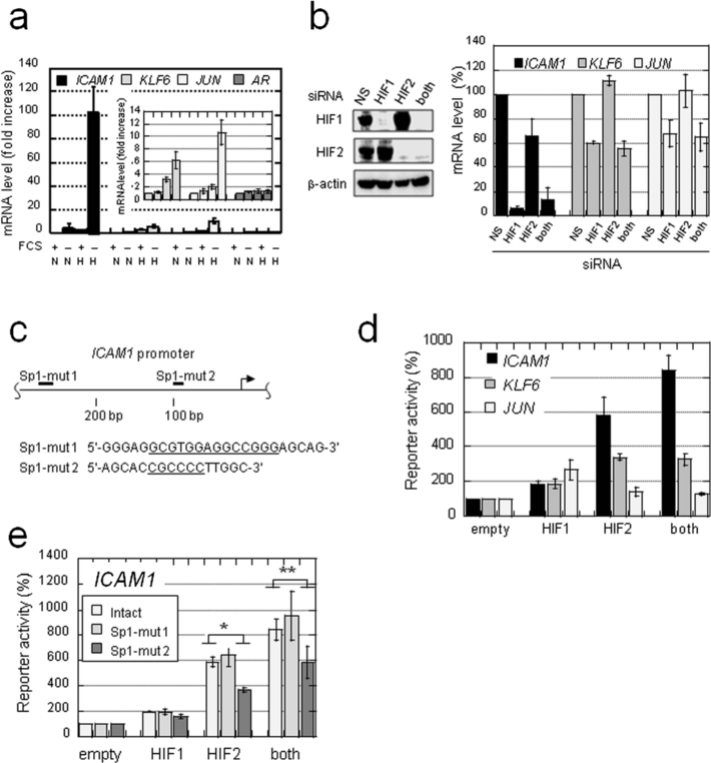
**Sp1 and HIFs contribute to the synergistic activation of multiple genes in OVSAYO cells under SSH. a)** Synergistic activation of genes under SSH. N and H indicate normoxia (20% O_2_ for 16 h) and hypoxia (1% O_2_ for 16 h), respectively. Data shown represent the mean (n = 3) ± SD. **b)** Activation of multiple genes under hypoxia is dependent on HIFs. Western blotting is also shown for HIFs. Data shown are the mean (n = 3) ± SD. **c)** The *ICAM1* promoter region and predicted Sp1 binding sites (ref. 19). PCR-amplicons generated in the reporter gene assay are shown in Additional file 2: Figure S1a. Bold bars: mutated sites (Sp1-mut 1 and 2); underlined nucleotide sequences: regions deleted by mutagenesis. **d, e)** Luciferase reporter gene assay. Plasmids were co-transfected with the indicated vectors, and luciferase activities were measured at 24 h post-transfection. Luciferase activities measured in cells transfected with the empty vector were defined as 100%. Data shown are the mean (n = 3) ± SD. *P = 0.0018; **P = 0.075.

**Figure 3:**
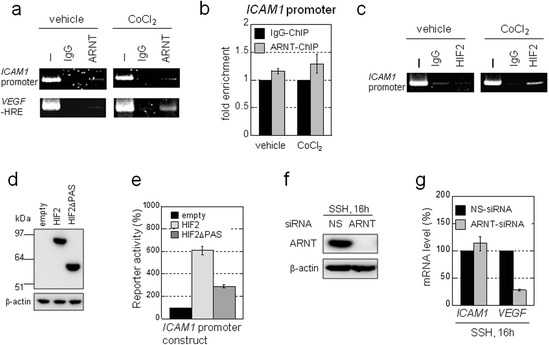
**Synergistic activation of the** ***ICAM1*** **gene is independent of ARNT. a)** ChIP analysis of ARNT binding to gene regulatory regions of the indicated genes. Cells were cultured for 4 h under stimulation with CoCl_2_ (500 μM) or treatment with a vehicle control. I: Input control lysate without immunoprecipitation. IgG: negative control using IgG. **b)** Results in a) were quantitatively estimated by real-time PCR analysis. **c)** ChIP analysis of HIF2 binding to the *ICAM1* promoter region. Cells were treated as in a). **d)** The *ICAM1* promoter construct (Figure 2e) was co-transfected with the indicated expression vectors. After 24 h, expression of indicated proteins was confirmed by western blotting. **e)** Luciferase activities were measured at 24 h post-transfection. Luciferase activities measured in cells transfected with the empty vector were defined as 100%. Data shown are the mean (n = 3) ± SD. **f)** Western blot analysis of lysates from OVSAYO cells transfected with non-specific (NS) or ARNT siRNA and then cultured under SSH for an additional 16 h. **g)** Real-time RT-PCR analysis of *ICAM1* and *VEGF* gene expressions in OVSAYO cells transfected with NS or ARNT siRNA and then cultured under SSH conditions for an additional 16 h. Data shown are the mean (n = 3) ± SD.

We tested whether genes activated in an Sp1-dependent manner were also inducible under real hypoxia (1% O_2_) by focusing on 4 genes (*ICAM1* [19], *KLF6* [20], *JUN* [21], and *AR* [22]; Figure 1b), as their upregulation by direct interactions with HIFs has not been reported and their basal promoter activities are regulated by Sp1. Real-time reverse-transcription polymerase chain reaction (RT-PCR) analysis revealed that *ICAM1*, *KLF6*, and *JUN* genes were weakly upregulated under hypoxia and in the presence of fetal calf serum (FCS) (Figure 2a, FCS+ H) compared with normoxic conditions (Figure 2a, FCS+ N). RT-PCR results also showed that siRNA-mediated knockdown of HIFs, particularly HIF1, impaired hypoxic induction of these genes (Figure 2b). We confirmed by chromatin immunoprecipitation (ChIP) that Sp1 and HIFs bind authentic promoter regions of these 3 genes in OVSAYO cells (Additional file 2: Figure S1a). Furthermore, luciferase reporter gene analysis revealed that the expression of HIFs can activate the *ICAM1* promoter more efficiently than the *KLF6* and *JUN* promoters (Figure 2c and d). Unlike knockdown experiments targeting endogenous genes, the effect on *ICAM1* promoter activation was predominant for HIF2. Further experiments demonstrated that activation of the *ICAM1* promoter by HIF2 is impaired when a known Sp1 binding site is mutated (Figure 2c and e). ChIP assay results showed that ARNT weakly bound to the *ICAM1* promoter region, but this binding was not enhanced in response to CoCl_2_ treatment (Figure 3a and b). However, HIF2 became bind the promoter region by CoCl_2_ exposure (Figure 3c). As expected, ARNT was found to bind the *VEGF*-HRE region in response to CoCl_2_ treatment (Figure 3a).

Previously, we demonstrated that the PAS domains of HIF2 are required for its association with Sp1 [12]. Thus, here we tested whether the PAS domains are important for activation of the *ICAM1* promoter by HIF2. Luciferase reporter gene analysis revealed that HIF2-driven activation of the *ICAM1* promoter was largely impaired when the domain-deleted HIF2 mutant (HIF2ΔPAS; 351–870 construct; ref. 12) was expressed instead of the full-length HIF2 (Figure 3d and e). Collectively, these results demonstrate that Sp1 and HIF2 can cooperate to mediate *ICAM1* induction in a HRE-independent manner.

### Sp1-dependent gene activation under hypoxia is synergistically enhanced by serum deprivation

Serum starvation is a crucial component of ischemia [23]. Hence, we next tested whether Sp1-dependent activation of the selected genes is enhanced under serum starvation and hypoxia (SSH), as in the case of *FVII* [12]. We found that *ICAM1*, *KLF6*, and *JUN*, but not *AR*, are synergistically activated under SSH. Notably, *ICAM1* expression was dramatically induced (Figure 2a, FCS– H). This robust expression under SSH may be characteristic of CCC cells as this was also true in an additional CCC cell line, OVISE (Additional file 2: Figure S1b). Other ovarian cancer cells of different histological origin did not exhibit such highly synergistic activation of genes (data not shown). We found that the synergistic *ICAM1* gene induction was decreased by Sp1 knockdown at mRNA (Additional file 2: Figure S2a) and protein (Additional file 2: Figure S2b) levels. This Sp1 dependence was also confirmed for *KLF6* and *JUN* induction, although such SSH-specific effects were not observed for *VEGF* expression (Additional file 2: Figure S2a).

The synergism involved in *ICAM1* induction was evident. It has been reported that overexpression of ICAM1 protein correlates with cancer prognosis [24]. Thus, we focused our study primarily on this gene. We found that synergistic *ICAM1* expression was decreased by silencing HIFs (Additional file 2: Figure S2c). However, unlike in hypoxic conditions with serum stimulation, both HIF1 and HIF2 contributed equally to synergistic *ICAM1* induction. Moreover, real-time RT-PCR analysis revealed that synergistic activation of the *ICAM1* gene under SSH conditions is not affected by ARNT knockdown, while *VEGF* expression was largely diminished (Figure 3f and g). These results demonstrate that *ICAM1* gene induction under SSH can be mediated *via* ARNT-independent interactions between Sp1 and HIFs within the gene promoter region.

### UPR does not contribute to synergistic *ICAM1* activation

A UPR marker CHOP is induced in OVSAYO cells cultured under SSH (Additional file 2: Figure S3a). Thus, we tested whether expression of the *ICAM1* gene under hypoxia with or without FCS treatment was enhanced by simultaneous treatment with the UPR-inducing agent, tunicamycin (Tun). Tun induced CHOP under hypoxia with or without FCS (Additional file 2: Figure S3b and c) and suppressed *ICAM1* expression (Additional file 2: Figure S3d and e). The expression levels of Sp1 and HIFs were not significantly altered (Additional file 2: Figure S3b), suggesting that the synergistic activation is not mediated by the UPR, as is the case with *FVII* [12].

### mTOR is involved in synergistic gene activation under SSH

**Figure 4:**
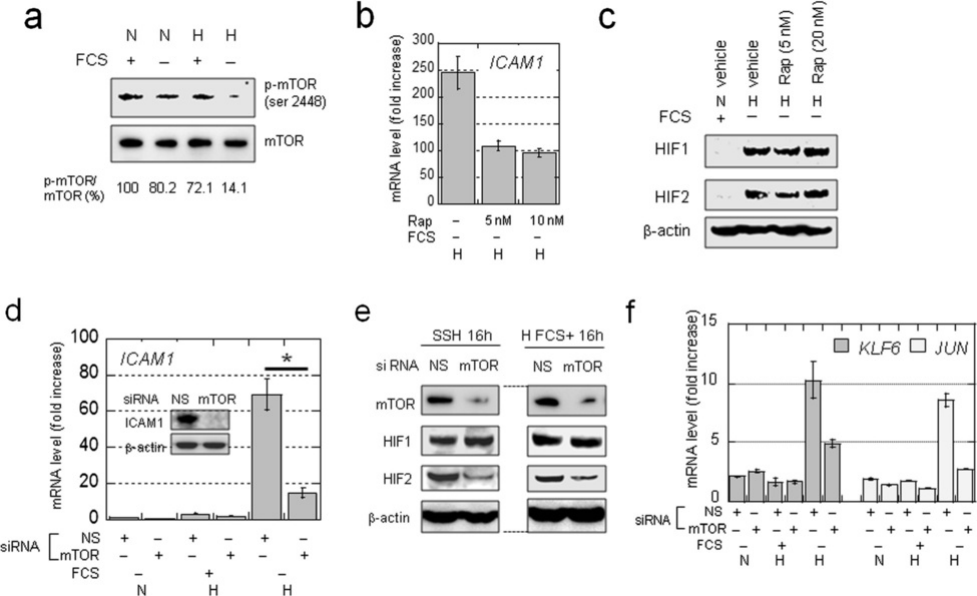
**Involvement of mTOR in the synergistic activation of** ***ICAM1*** **. a)** Western blot analysis of mTOR phosphorylation levels in OVSAYO cells cultured under the indicated conditions for 16 h. The phosphorylation levels shown were based on band intensity quantification using ImageJ software. **b)** Effect of rapamycin on the synergistic activation of *ICAM1* in cells cultured under SSH for 16 h. Data shown are the mean (n = 2) ± SD. **c)** Western blot analysis of HIF expression in OVSAYO cells cultured under SSH for 16 h in the presence or absence of rapamycin. **d)** Effect of mTOR on the synergistic *ICAM1* activation in OVSAYO cells transfected with siRNAs. Data shown are the mean (n = 3) ± SD. *P = 0.019, compared with the vehicle control. Inset: western blot analysis of ICAM1 expression in cells transfected with the indicated siRNAs and cultured under SSH for 24 h. **e)** Effects of mTOR on the expression levels of HIFs in OVSAYO cells cultured as indicated. **f)** Effects of mTOR on mRNA levels of *KLF6* and *JUN* in OVSAYO cells. Cells were transfected with siRNAs as indicted and cultured for 24 h. Cells were further cultured as indicated for 16 h. Data shown are the mean (n = 2) ± SD.

Western blotting showed that mTOR activation was suppressed in OVSAYO cells cultured under SSH, as phosphorylation at Ser2448 was decreased compared that observed under non-SSH conditions (Figure 4a). We next tested the requirement of mTOR for synergistic gene activation, as the active mTOR fraction remaining may function under SSH. Real-time RT-PCR revealed that *ICAM1* activation under SSH conditions in OVSAYO cells was diminished by treatment with the mTOR inhibitor rapamycin (Rap), which predominantly inhibits mTORC1 [5] (Figure 4b). The expression of HIFs was not influenced by Rap treatment (Figure 4c), suggesting that the effect of Rap is not due to translational inhibition of HIFs. We also confirmed that synergistic *ICAM1* activation was decreased by mTOR silencing at mRNA and protein levels (Figure 4d and e). Notably, unlike Rap treatment, HIF2 under SSH (but not HIF1) was downregulated when mTOR was silenced (Figure 4e), implying that mTORC2 is responsible for HIF2 translation [6]. However, this decrease was not responsible for inhibition of synergistic *ICAM1* activation, as reduction of HIF2 levels was also seen in cells cultured with FCS (Figure 4e).

We tested the effect of mTOR on the synergistic activation of other genes. Real-time RT-PCR revealed that *KLF6* and *JUN* induction was also repressed by mTOR silencing under SSH (Figure 4f). Furthermore, Sp1 levels in OVSAYO cells were not significantly affected by changes in the culture conditions (Additional file 2: Figure S4a). Thus, the effect of mTOR is independent of HIFs and Sp1 levels.

### Activation of NFκB is crucial for synergistic *ICAM1* induction

**Figure 5:**
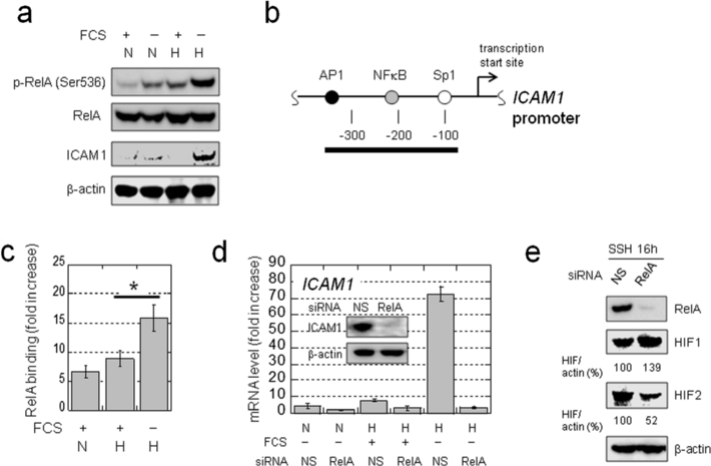
**NFκB activation is essential for synergistic** ***ICAM1*** **activation in OVSAYO cells. a)** Western blot analysis of phospho-RelA (Ser536) or total RelA expression following ICAM1 induction in cells cultured under the indicated conditions for 16 h. N and H indicate normoxia (20% O_2_) and hypoxia (1% O_2_), respectively. **b)** Diagram of the *ICAM1* promoter region and the positions of the Sp1, NFκB, and AP1 binding sites. A black bar indicates the PCR amplicon for the ChIP assay. **c)** Quantitative ChIP analysis of RelA binding to the *ICAM1* promoter region by real-time PCR, using cells cultured for 16 h under the indicated conditions. RelA binding levels are represented as fold-increases relative to negative control experiments using IgG. Data shown are the mean (n = 3) ± SD. *P = 0.02. **d)** Effect of RelA on synergistic *ICAM1* activation in cells cultured for 16 h under the indicated conditions. Data are the mean (n = 3) ± SD. Inset: western blot analysis of ICAM1 expression in cells transfected with indicated siRNAs and cultured under SSH for 24 h. **e)** Western blotting against RelA and HIFs in lysates from cells transfected with the indicated siRNAs. HIF levels shown are based on band intensities quantified using ImageJ software.

NFκB is activated during inflammation, hypoxia, and irradiation and induces the expression of a variety of genes, including *ICAM1* [19,25-28]. Moreover, NFκB enhances HIF1 expression at the transcriptional level [29]. However, it is unclear whether NFκB contributes to the unusual synergism of *ICAM1* activation under SSH. We found that the RelA subunit of NFκB became highly phosphorylated under SSH in association with SSH-specific ICAM1 induction (Figure 5a). Western blotting of cytoplasmic and nuclear fractions revealed that RelA was translocated from the cytoplasm to the nucleus under SSH, subsequent to the expression of HIFs (Additional file 2: Figure S4a). ChIP assays demonstrated that NFκB binding to the *ICAM1* promoter (Figure 5b) is highest under SSH (Figure 5c), and real-time RT-PCR revealed that *ICAM1* expression at mRNA and protein levels under SSH was markedly decreased by RelA knockdown (Figure 5d). The relative expression pattern of HIFs can be changed by RelA silencing (Figure 5e). Thus, NFκB may influence *ICAM1* expression through altered HIF expression pattern. In contrast, experiments with the same RNA samples showed that *KLF6* and *JUN* expression under SSH was not influenced by RelA silencing (Additional file 2: Figure S4b).

### NFκB activation under SSH involves autonomous TNFα production

**Figure 6:**
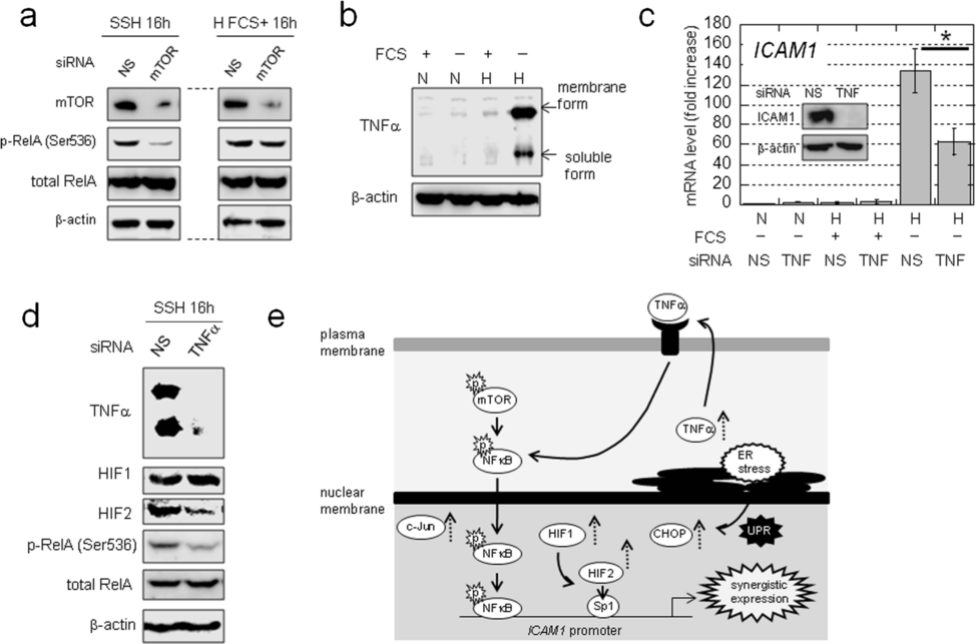
**NFκB activation is associated with mTOR and autonomously-produced TNFα. a)** Effect of mTOR on RelA activation in OVSAYO cells cultured under hypoxia. **b)** TNFα production in OVSAYO cells cultured under SSH for 24 h. **c)** Effect of TNFα on synergistic *ICAM1* activation in cells transfected with siRNAs, as indicated. Data shown are the mean (n = 3) ± SD. *P = 0.018. Inset: western blot analysis of ICAM1 expression in cells transfected with the indicated siRNAs and cultured under SSH for 24 h. **d)** Effect of TNFα on RelA activation under SSH in OVSAYO cells. **e)** Model of *ICAM1* induction under SSH. Solid arrows, dashed arrows, and “p”s with bursts are indicative of the activation process, protein induction, and active (phosphorylated) states, respectively.

We next investigated the mechanisms involved in NFκB activation under SSH. We first evaluated the effect of mTOR, as synergistic *ICAM1* activation is partly dependent upon this kinase activity. Western blotting showed that RelA phosphorylation under SSH was decreased by mTOR knockdown (Figure 6a). In contrast, phosphorylation levels under hypoxia with concurrent FCS treatment were not influenced by mTOR knockdown (Figure 6a), suggesting that mTOR associates with NFκB activity under SSH.

To explore the mechanisms of NFκB activation, we performed cDNA microarray analysis of genes synergistically activated under SSH. We found the ratio of genes with increased expression under SSH to that observed under normoxia with FCS was > 3, while this ratio was < 2 when comparing gene induction under normoxia without FCS and hypoxia with FCS (Additional file 2: Figure S4c and Additional file 3: Table S2). To identify potential mechanisms for these observations, we performed pathway analyses of these genes using the Kyoto Encyclopedia of Genes and Genomes database. We found that in addition to *ICAM1*, tumor necrosis factor-α (TNFα) is synergistically upregulated under SSH (Additional file 2: Figure S4d) and could potentially activate NFκB. We found that TNFα protein levels were low under normoxia and hypoxia with FCS stimulation, but were robustly increased in OVSAYO cells cultured under SSH (Figure 6b).

Exposure to exogenous TNFα can induce *ICAM1* in cancer cells [27,30]. However, it is unclear how self-produced TNFα affects SSH-specific gene activation in CCC cells. Thus, we examined the effect of autonomously produced TNFα on the synergistic activation of *ICAM1* in OVSAYO cells by real-time RT-PCR. Synergistic *ICAM1* activation was reduced (Figure 6c) in OVSAYO cells in which TNFα expression was completely blocked by RNAi (Figure 6d). Notably, RelA phosphorylation levels under SSH were reduced by TNFα knockdown (Figure 6d), although phosphorylation under hypoxia plus FCS stimulation was not (Additional file 2: Figure S4e). The amount of HIF2 (but not HIF1) induced by SSH or hypoxia plus FCS was decreased by TNFα silencing (Figure 6d and Additional file 2: Figure S4e). Taken together, these data indicate that mTOR expression may correlate with TNFα under SSH.

We additionally tested whether synergistically induced c-Jun expression contributes to the robust expression of ICAM1, as c-Jun can regulate the *ICAM1* promoter (Additional file 2: Figure S5a). Various experiments demonstrated that c-Jun does not contribute to synergistic *ICAM1* activation (Additional file 2: Figure S5). Thus, we can summarize *ICAM1* induction under SSH, based on mechanisms described above (Figure 6e).

### Serum factors responsible for synergistic gene activation

**Figure 7:**
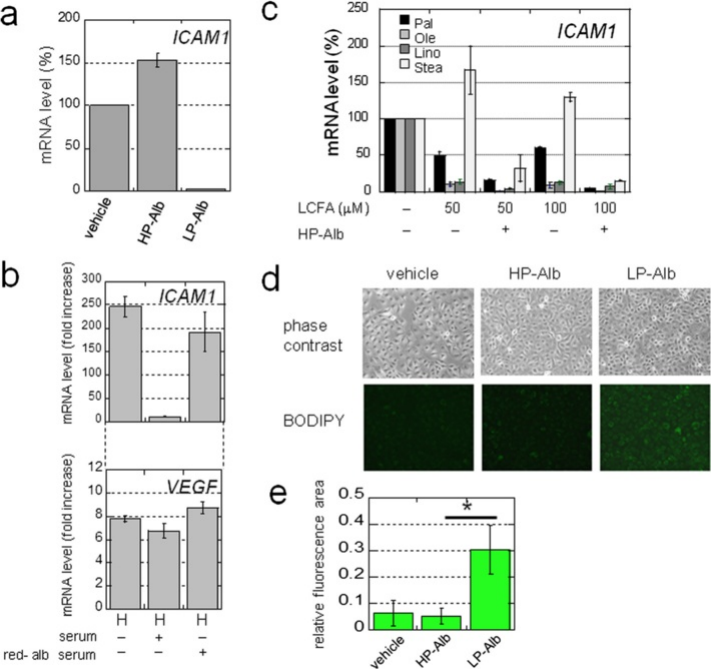
**LCFAs are responsible for the synergistic activation of** ***ICAM1*** **. a)** mRNA levels of *ICAM1* in OVSAYO cells cultured under SSH for 16 h with HP- or LP-Alb. Data shown are the mean (n = 2) ± SD. **b)** *ICAM1* and *VEGF* mRNA expression levels in cells cultured with 1% serum for 16 h. N, normoxia (20% O_2_); H, hypoxia (1% O_2_). Data shown are the mean (n = 3) ± SD. **c)** *ICAM1* expression in cells cultured as in **a)** in the presence or absence of HP-Alb and various LCFAs. Data shown are the mean (n = 2) ± SD. **d)** Staining of neutral lipids in OVSAYO cells cultured as in **a)** with or without albumin. **e)** Quantitative representation of **d)**. Fluorescence areas were quantified using ImageJ software. Data shown are the mean (n = 6) ± SD. *P = 0.0002.

An unanswered question is the identity of serum factors that are responsible for synergistic gene activation. We initially found that the addition of growth factors does not suppress synergistic *ICAM1* activation (data not shown). Thus, we tested whether synergistic *ICAM1* induction could be repressed by the addition of albumin to serum-free culture media, as albumin is the most abundant human plasma protein and plays multiple roles in regulating blood functions [31]. We used an albumin concentration of 44 μM, which corresponds to protein level found in culture medium (2.9 mg/ml) with 10% FCS. We observed by real-time RT-PCR that *ICAM1* mRNA levels under SSH were dramatically reduced in cells cultured with bovine serum albumin compared with those found without albumin (data not shown). We repeated these experiments with human albumin of differing purities. Quantitative RT-PCR results revealed that synergistic *ICAM1* activation was markedly decreased in cells cultured with low purity (LP) albumin (Figure 7a). However, *ICAM1* induction in cells cultured with highly purified (HP) albumin did not decrease compared with the vehicle control (Figure 7a). This albumin-dependent *ICAM1* induction was also observed by RT-PCR using reduced-albumin (red-alb) serum (Additional file 2: Figure S6a and Figure 7b). However, parallel expression differences were not observed for *VEGF* (Figure 7b). These results suggested that LP-albumin contains serum factors responsible for suppression of the synergistic *ICAM1* activation. We further showed that synergistic activation of the *KLF6* and *JUN* genes exhibits a similar expression pattern in response to red-alb serum (Additional file 2: Figure S6b).

### Long chain fatty acids are responsible for the suppression of synergistic gene activation under SSH

Plasma albumin binds multiple molecules including nutrients, hormones, and metabolites [31,32]. We next examined the effect of long chain fatty acids (LCFAs) on synergistic *ICAM1* activation as the HP-albumin used in this study is devoid of fatty-acids. Real-time RT-PCR analysis revealed that a physiological concentration of unsaturated LCFAs [33] (oleic and linoleic acids) largely suppressed *ICAM1* induction under SSH in OVSAYO cells (Figure 7c). This decrease was further enhanced in the presence of HP-albumin (Figure 7c). Weak or no suppression of *ICAM1* expression was observed after exposing cells to physiological levels of saturated LCFAs (palmitic and stearic acids) alone (Figure 7c). However, *ICAM1* induction was considerably suppressed when cells were simultaneously cultured with these LCFAs and HP-albumin.

We next determined the effect of LP- and HP-albumin treatment on lipid levels in cells cultured under SSH as LCFA uptake results in cytoplasmic lipid droplet accumulation in cancer cells [34]. To examine the cellular neutral lipid levels, cells were cultured in serum-free medium in the presence or absence of LP- and HP-albumin, and then stained with a neutral lipid marker, the BODIPY fluorescent dye [34]. We detected stronger BODIPY fluorescence when cells were cultured with LP-albumin (Figure 7d and e), suggesting that cells are starved of neutral lipids when cultured without LCFA. This conclusion is supported by the detection of upregulated of enhanced fatty acid synthase (Additional file 2: Figure S6c), as expression of this enzyme is increased in response to cellular lipid deficiency [35].

### Effect of ICAM1 on CCC cell phenotype

**Figure 8:**
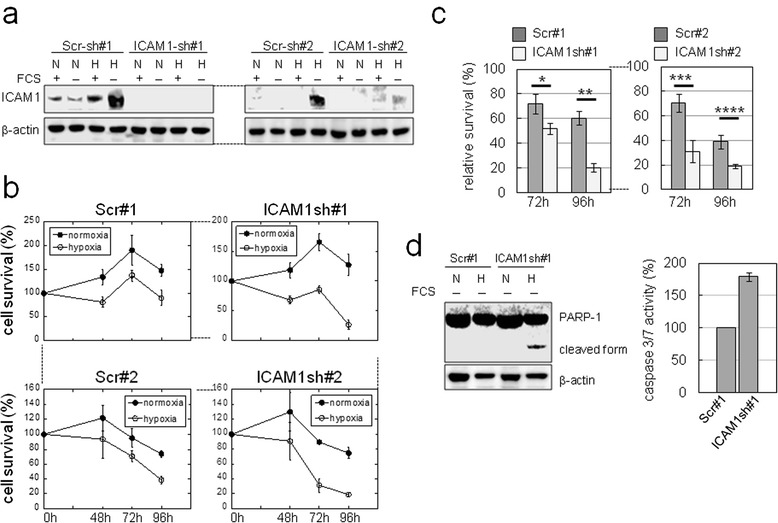
**ICAM1 confers a survival advantage to CCC cells. a)** Western blot analysis of ICAM1 expression in OVISE cells transfected with shRNAs. N, normoxia for 24 h; H, 1% O_2_ for 24 h. **b)** MTS assays revealed the viability of OVISE cells cultured under serum-free conditions. **c)** Relative survival rates of OVISE cells cultured as in **b)**. The viabilities of cells cultured under SSH (estimated as in **b)** were normalized to viability rates observed with cells cultured under normoxia without FCS. Data shown are the mean (n = 3) ± SD. *P = 0.036; **P = 0.001; ***P = 0.009; ****P = 0.007. **d)** Left: western blot of PARP-1 in OVISE cells. N, normoxia for 72 h; H, 1% O_2_ for 72 h. Right: caspase activities in OVISE cells cultured under SSH for 96 h.

ICAM1 expression within poorly vascularized necrotic regions was observed in clinical CCC samples (Additional file 2: Figure S7). Thus, we next examined whether ICAM1 confers a survival advantage to ovarian cancer cells under SSH. Cloned OVISE cells stably transfected with scrambled control (Scr-) or ICAM1-shRNA were prepared. The loss of ICAM1 induction in OVISE cells under SSH was confirmed by western blotting (Figure 8a). We found that the viability of ICAM1-silenced cells under SSH was more significantly reduced compared to control shRNA cells (Figure 8b and c). Similar results were obtained in experiments using cell clones derived from OVSAYO cells (Additional file 2: Figure S8). Thus, ICAM1 confers a survival advantage to CCC cells exposed to SSH.

We performed western blot analysis of PARP-1 expression to ascertain whether ICAM1 can inhibit apoptotic cell death. We detected the cleaved form of PARP-1 only in ICAM1-shRNA-transfected OVISE cells cultured under SSH (Figure 8d). We next examined caspase activities in shRNA-transfected cells cultured under the same SSH conditions. We found that the activities of caspases-3 and -7 were elevated in ICAM1-shRNA-transfected cells compared with control shRNA cells (Figure 8d). These results suggested that ICAM1 confers an anti-apoptotic effect under SSH.

### ICAM1 is induced *in vivo* and confers tumor growth advantage

**Figure 9:**
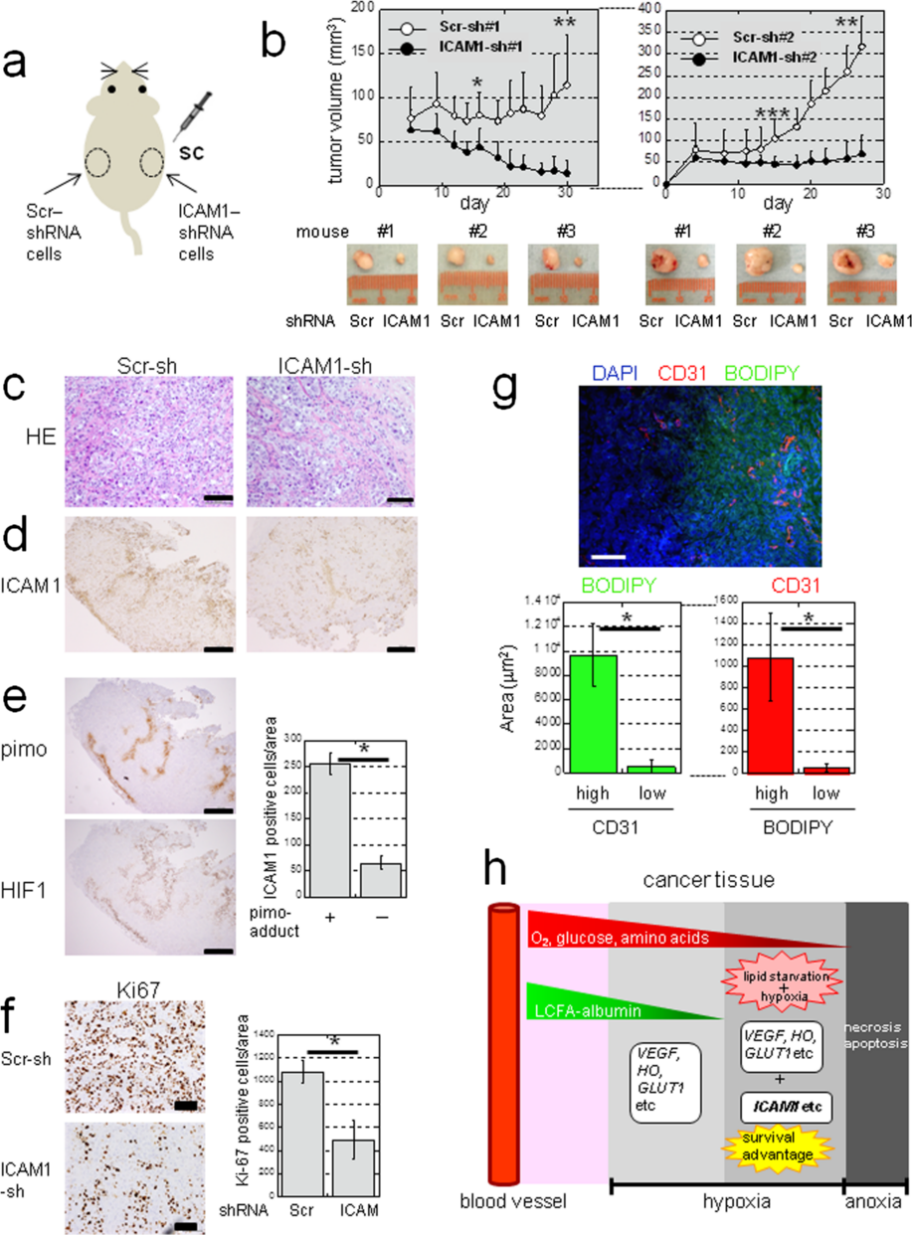
**ICAM1 is inducible** ***in vivo*** **and confers a growth advantage to CCC tumors. a)** Sites where NOD-SCID mice were injected with cells expressing Scr-shRNA (#1 or #2) or ICAM1-shRNA (#1 or #2). **b)** Tumor volumes in mice injected with Scr-shRNA #1 or ICAM1-shRNA #1 (n = 9) or Scr-shRNA #2 or ICAM1 shRNA #2 (n = 10). *P = 0.0006; **P < 0.0001; ***P = 0.002. **c)** H&E staining (scale bar: 100 μm). Immunohistochemistry of ICAM1 **d)** Pimo-adduct and HIF1 **e)**. Quantitation of ICAM1-positive cells within pimo (+) or (−) areas (n = 8). *P < 0.0001. Scale bar: 500 μm. **f)** Immunohistochemistry of Ki67. Scale bar: 100 μm. Quantitation of Ki67 positive cells is shown (n = 8). *P < 0.0001. **g)** Lipid staining using frozen sections of tumor tissue. Immunofluorescence of CD31 was also performed for vessels. Scale bar: 100 μm. The relationship between neutral lipid staining with BODIPY and CD31 positivity was quantified (n = 10). *P < 0.0001. **h)** Hypothetical model of lipid starvation and hypoxia associated with induction of genes of different categories in tumor tissues. Diffusion of LCFAs associated with high molecular weight albumin from the blood stream is limited compared to that of smaller molecules (O_2_, glucose, and amino acids). Unlike (*VEGF*), (*HO*) and (*GLUT1*) genes, Sp1-dependent genes such as *ICAM1* may be efficiently up-regulated only under hypoxia with a limited LCFA supply, resulting in a survival advantage of cancer cells to such a severe microenvironment.

Some studies have shown that ICAM1 expression impairs progression of non-CCC ovarian cancers [30,36]. Thus, we further explored whether ICAM1 is induced *in vivo* and if so, how ICAM1 expression affects tumor growth. We first examined the proliferation of OVISE cells stably transfected with Scr-shRNAs (Scr#1 and #2) or ICAM1 shRNAs (ICAM1#1 and #2) under normoxia plus FCS stimulation and confirmed that ICAM1 knockdown caused no significant effects (Additional file 2: Figure S9a). We next subcutaneously injected Scr- and ICAM1-shRNA transfected cells into NOD-SCID mice (Figure 9a) and monitored subsequent tumor growths. We found that the growth of xenograft tumors derived from ICAM1-shRNA-transfected cells was inhibited compared with that of control cells (Figure 9b). Western blotting showed that ICAM1 levels in control tumors were higher than those of tumors with ICAM1-shRNA (Additional file 2: Figure S9b).

Hematoxylin and eosin (H&E) staining revealed tumor tissues with typical CCC histological patterns of clear cytoplasmic and dark nuclear staining (Figure 9c). Immunohistochemistry (IHC) revealed that ICAM1 staining was stronger in control tumors than in ICAM1-silenced tumors (Figure 9d). IHC using antibodies against pimonidazole-adducts (a severe hypoxia marker, hereafter referred to as pimo-adducts) and a milder hypoxia marker (HIF1 and HIF2) revealed that the cell surface-staining pattern of ICAM1 expression (Figure 9d, e, and Additional file 2: Figure S10a) was clearly stronger within severely hypoxic areas (Figure 9e). *In situ* hybridization experiments provided further evidence of ICAM1 mRNA induction within severely hypoxic regions of the control tumors (Additional file 2: Figure S10b). However, *ICAM1* transcripts were not detected within ICAM1-silenced tumors, although these tumor tissues were also hypoxic (Additional file 2: Figure S10c).

IHC staining further revealed that the number of Ki67-positive cells within control tumors was greater than that in ICAM1-silenced tumors (Figure 9f). However, Ki67-positive cells were poorly detected within ICAM1-positive areas within tumor tissues (Additional file 2: Figure S10d and e), suggesting that ICAM1 indirectly contributes to tumor growth possibly by enhancing the viability of cells.

Lipid and vessel staining revealed that tissue lipid levels were lower in vessel-poor regions in ICAM1-positive xenograft tumor tissues (Figure 9g and Additional file 2: Figure S11a), suggesting a directly proportional correlation between lipid contents and vessel densities. Immunofluorescence staining further showed that ICAM1 was strongly expressed within tissues in severely hypoxic regions (Additional file 2: Figure S11b).

## Discussion

Here, we demonstrated that Sp1 mediates the activation of multiple genes in cooperation with HIFs. This transcriptional activation is synergistically enhanced under SSH, in contrast to the activation of authentic HIF-dependent genes. We further showed that an insufficient supply of LCFAs can lead to synergistic gene activation. The ICAM1 protein is dramatically induced by this mechanism and is critical for tumor growth *in vivo*, thus indicating a novel mechanism for adaptation to LCFA starvation and hypoxia as a means to promote tumor growth.

Evidence presented in this study suggests that direct binding of both HIFs and NFκB to the *ICAM1* promoter region is essential for synergistic *ICAM1* activation. Notably, HIF2 binding appears to be mediated via interaction with Sp1. The synergistic activation of *KLF6* and *JUN* occurs independently of NFκB. This suggests that transcriptional cooperation between Sp1, HIFs, and NFκB promotes rigorous *ICAM1* upregulation under lipid starvation and hypoxia through a distinct mechanism. In addition, the question arises as to how NFκB may be activated under SSH, a condition where exogenous stimuli such as cytokines are absent. Our study provides 2 answers to this question. First, the activation is associated with mTOR activity. This may correlate with earlier observations that, under non-hypoxic conditions, mTOR can cause NFκB activation, leading to *ICAM1* induction in cancerous and normal cells [25,28]. Second, NFκB can be activated by autonomously produced TNFα in an autocrine manner.

Recent evidence has shown that cancer cells utilize aberrant mechanisms for energy metabolism [37]. Some cancer cell types predominantly consume fatty acids rather than glucose, relying on β-oxidation for energy production [34,35]. In addition to their normal role as an energy source, recent studies have revealed that LCFAs may be involved in the tolerance of cancer cells to various cellular stresses [35,38]. LCFAs exist in the bloodstream in complex with albumin. It is possible that the cellular uptake of LCFAs may be facilitated only in association with albumin, resulting in their conversion to neutral lipids (acyl-CoA derivatives) [39] in CCC cells. In the present study, we showed that the removal of LCFAs synergistically enhances Sp1-dependent activation of multiple genes under hypoxia. This effect appears to be greater for unsaturated LCFAs than for saturated LCFAs. This difference may be attributed to their roles in transcriptional regulation in addition to altered metabolism pathways among LCFAs, as saturated and unsaturated LCFAs could be differentially utilized as ligands for some nuclear receptors [40]. It is necessary to uncover the detailed mechanism(s) by which CCC cells sense LCFA-starvation and hypoxia to trigger cellular signaling and induce unusually high gene expression.

We found that ICAM1 enhances cell survival under SSH at least in part through the inhibition of apoptosis. This is distinct from the survival mechanism whereby unsaturated LCFAs protect against UPR-dependent cell death following ischemic conditions mediated by mTOR activity [38]. Furthermore, ICAM1 promotes CCC tumor growth in mice. Previous reports have found that ICAM1 induction during inflammation enhances eosinophil survival by activating cellular signaling mechanisms, resulting in eosinophilia in asthma [41]. Thus, CCC cells might similarly activate signaling cascades via ICAM1, enabling cells to survive under severely hypoxic conditions. In additional, ICAM1 is known to be overexpressed in cancer cells where it recruits immune cells to promote tumor progression [42]. Thus, immune cell-mediated mechanisms may contribute to the acceleration of tumor growth, although it is not clear to what extent immune cells contributed in our immunodeficient mouse model. Furthermore, our data contradict earlier reports showing that ICAM1 suppresses ovarian cancer progression [30,36]. These discrepancies may be due to different histological types or experimental settings tested.

The severity of hypoxia within tumor tissues varies depending on the sparsity and aberrant nature of local vasculatures, thus creating microenvironments with limited gradients of molecules supplied from the blood. Diffusion-limited hypoxia occurs at ~100-μm distances from blood vessels, and hypoxic severity in tissues increases with increasing distances [43]. Given that the sizes of O_2_, glucose, and amino acids are smaller than that of LCFA (in complex with albumin), it is likely that areas with lipid starvation, hypoxia, expression of HIFs, and a nutrient supply exists in tumor tissues (Figure 9h). In this case, tissue lipid levels may be used to define a more severe hypoxia *via* Sp1 (Figure 9h). It was recently reported that immunotherapy targeting ICAM1 is effective in treating multiple myeloma [44]. Thus, further clinical investigations will likely support the validity of lipid starvation and hypoxia as a target of cancer therapy.

## Methods

### Reagents

The following reagents were obtained from Sigma (St. Louis, MO, USA): tunicamycin (T7765), HP-albumin from human serum, fatty acid-free (A3782), LP-albumin from human serum (A1653), palmitic acid (P0500), oleic acid (O1008), linoleic acid (L1376), and stearic acid (S4751). Rapamycin (553210) was purchased from (Millipore, Temecula, CA, USA).

### Cell culture

Human cancer cell lines used in this study were cultured as previously described [8].

### Transfection with expression vectors and siRNAs

Expression vectors and siRNAs targeting Sp1 and HIFs were described previously [12]. We employed 2 individual siRNA sequences targeting each target protein. See Additional file 4 for siRNAs.

### cDNA microarray analysis

Total RNA was isolated as previously described [8]. cDNA microarray analysis of genes activated in response to CoCl_2_ stimulation in an Sp1-dependent manner was performed by Takara (Shiga, Japan), using a human whole genome array (Agilent Technologies, Santa Clara, CA, USA). The effects of serum deprivation on the expression of genes under normoxic and hypoxic conditions were analyzed by cDNA microarray analysis using a NimbleGen Catalog Eukaryotic Gene Expression 385 K Array (#5543886, Roche, Madison, WI, USA). Arrays were scanned using a GenePix 4100A Microarray Scanner (Axon Instruments, Union City, CA, USA), and data were analyzed by Subio, Inc. (Kagoshima, Japan).

### Real-time RT-PCR analysis

mRNA levels were determined as previously described [8,12]. See Additional file 4 for primer and probe sequences. The mRNA expression levels of 18S ribosomal RNA were measured using SYBR green-based detection [45].

### Protein quantification

Protein levels were quantified using the Micro BCA Protein Assay Kit (23235; Thermo Scientific, Rockford, IL, USA).

### Western blotting

Western blotting was performed using Novex NuPAGE 4–12% Bis-Tris gels (Life Technologies, Carlsbad, CA, USA). Detection was performed using ECL-Prime or ECL-Advance (GE Healthcare, Buckinghamshire, UK). See Additional file 4 for a description of the antibodies used.

### Reporter gene assays

Reporter gene assays were performed as previously described [12]. Plasmid constructs encoding the *ICAM1*, *KLF6*, and *JUN* 5′promoter regions used in ChIP assays were prepared as previously described [12]. See Additional file 4 for more details.

### ChIP assays

ChIP analyses were performed primarily as previously described [8,12]. See Additional file 4 for more details.

### Preparation of cytoplasmic and nuclear fractions

Cytoplasmic and nuclear fractions were prepared using an NE-PER Nuclear and Cytoplasmic Extraction Reagents Kit (Thermo Scientific).

### Preparation of reduced-albumin serum

Albumin was removed from human serum (Takara Bio, Shiga, Japan) using a Proteoprep Immunoaffinity Albumin & IgG Depletion Kit (Sigma).

### Preparation of cells stably transfected with small hairpin RNAs

Cells were stably transfected with short hairpin RNAs (shRNAs) using pcDNA6.2-GW/miR, pLenti6/V5-DEST, and the ViraPower Lentiviral Expression System (Life Technologies). Several independent clones were isolated by selecting cells in 10 μg/mL blasticidin. See Additional file 4 for the shRNA sequences used.

### Cell viability and caspase assays

Cell viabilities were estimated in MTS assays using the CellTiter96 Aqueous One Solution Cell Proliferation Assay (Promega, Madison, IL, USA). Caspase activities were determined by performing Caspase-Glo assays (Promega).

### Xenograft tumor experiments

The institutional review board at the Kanagawa Cancer Center Research Institute approved this study. OVISE (1.5 × 10^7^ cells) cell clones were subcutaneously injected into 9 (for Scr-shRNA or ICAM1 shRNA #1) and 10 (for Scr-shRNA or ICAM1 shRNA sh#2) NOD-SCID mice (Charles River Laboratories Japan Inc., Yokohama, Japan). Tumor growths were monitored by calculating tumor volumes as the long diameter × (short diameter)^2^ × 1/2. A Hypoxyprobe-1 Kit (Natural Pharmacia International, Burlington, MA, USA) was used to detect tissue hypoxia. Prior to tumor isolation, mice were injected intraperitoneally with pimonidazole-HCl solution (60 mg/kg). At 1 h post-injection, mice were sacrificed under general anesthesia with isoflurane, and tumors were isolated for further experiments.

### Immunohistochemistry and immunofluorescence

Routinely processed formalin-fixed, paraffin-embedded specimens were sectioned (4 μm thickness) and stained with antibodies. Immunoreactivity was visualized by the peroxidase-labeled amino acid polymer method, using Histofine Simple Stain MAX-PO (Nichirei Co., Tokyo, Japan) and the avidin-biotin-peroxidase complex method (LSAB+; DakoCytomation Co., Tokyo, Japan), according to the manufacturer’s instructions. Sections were counterstained with H&E. In the case of ICAM1 immunofluorescence, a secondary antibody (Alexa Fluor 488 anti-mouse IgG, Life Technologies) was used. Sections were counterstained with DAPI using Prolong Gold Antifade Reagent with DAPI (Life Technologies). Antibodies against the following targets were used for IHC at the indicated concentrations: ICAM1 (sc-8439, 4 μg/mL; Santa Cruz Biotechnology), pimo-adduct (Hypoxyprobe-1 Kit, 2.3 μg/mL; Hydroxyprobe), CD31 (6.7 μg/mL; Dianova GmbH, Hamburg, Germany), HIF-1α (2.5 μg/mL; BD Biosciences, San Jose CA, USA), HIF-2α (10 μg/ml; NB100-122, Novus Biologicals, Littleton, CO), and Ki67 (1:25 dilution; BD Biosciences).

### Microscopic detection of neutral lipid in cultured cells and tumor tissues

Neutral lipid in cultured cells and tumor tissues was detected by fluorescence microscopy using BODIPY lipid probes (Life Technologies). Cultured OVSAYO cells were washed twice with serum-free culture media and treated with 25 μM of BODIPY lipid probes in serum-free media for 5 min in a 37°C incubator. Cells were then washed twice with serum-free media and used for fluorescence detection. Images were acquired on an OLYMPUS CKX41 microscope. Fluorescence areas were quantified using ImageJ software (http://rsb.info.nih.gov/ij/). For tumor tissues, frozen sections (8-μm thickness) of xenograft tumors were treated with 100 μM of BODIPY lipid probes (Life Technologies) in 50 mM Tris-HCl (pH 7.6) for 30 min at ambient temperature. Tissues were then washed twice with 50 mM Tris-HCl (pH 7.6), and fluorescence detection was performed. Images were acquired on a BZ-9000 fluorescence microscope (Keyence, Osaka, Japan). Quantitative analysis of images was performed using equipped BZ-Analyzer software (Keyence).

### Antibody labeling

Labeling of an anti-pimo antibody with Alexa Fluor dye was performed using Zenon Mouse IgG Labeling Kits (Life Technologies), according to manufacturer’s recommended procedure.

### In situ hybridization

*In situ* hybridization was performed using an RNAscope 2.0 FFPE Assay Kit (Advanced Cell Diagnostics, Hayward, CA, USA) and an ICAM1 probe (402951, Advanced Cell Diagnostics). Routinely processed formalin-fixed paraffin-embedded specimens were sectioned (4-μm thickness) and used in this study.

### Clinical samples

FFPE tissues from surgically removed specimens were prepared from patients of the Yokohama City University Hospital under written agreements in the study, which was approved by our institutional review board.

### Statistics

Statistical significances were evaluated using 2-sided Student’s *t*-tests for 2 data sets. *P* values < 0.05 were considered statistically significant.

### Accession numbers

The GEO database accession number for the microarray dataset is GSE55565.

## Additional files

Additional file 1: Table S1. Full list of genes identified by microarray analysis described in Figure 1b. Genes appearing in this figure are underlined. Additional file 2: Figure S1. a) ChIP analysis. b) *ICAM1* expression in OVISE cells. **Figure S2.** a) Real-time RT-PCR analysis of *genes* in OVSAYO cells. b) Western blotting with OVSAYO cell lysates. c) Real-time RT-PCR analysis using cells cultured under SSH. Western blotting was also performed. **Figure S3.** a,b,c) Protein levels in OVSAYO cells. d) mRNA levels were analyzed for cells cultured with or without Tun. V: vehicle. e) Effect of Tun on the *ICAM1*expression. **Figure S4.** a) Western analysis of indicated fractions of OVSAYO cells. b) Effect of RelA on the activation of genes. c) Venn diagram representation of genes. d) Heat map representation of gene expressions. e) Western analysis of cells transfected with siRNA. **Figure S5.** a) AP1 and NFκB bind to the promoter region. b) Quantitative ChIP analysis of c-Jun binding to the *ICAM1* promoter. c) c-Jun expression in OVSAYO cells. d) Real-time RT-PCR analysis. Cells transfected with siRNAs were cultured under SSH. c-Jun expression in siRNA transfected cells is also shown. **Figure S6.** a) Approximately 80% of albumin was removed from human serum, resulting in reduced-albumin serum. b) Real-time RT-PCR analysis of indicated *genes*. c) Expression of fatty acid synthase (Fasn) in OVSAYO cells. **Figure S7.** a) ICAM1 immunocytochemistry in OVISE cells transfected with Scr- and ICAM1-shRNA #1. Cells were cultured under SSH. b) H&E staining and IHC using a surgically removed specimens from CCC patients. **Figure S8.** a) Western blot analysis, b,c) MTS assay. **Figure S9.** a) Proliferation of OVISE cells transfected with shRNAs. b) ICAM1 expression in tumor tissues. **Figure S10.** a,c,d) IHC using tumors transfected with Scr-shRNA#2. *In situ* hybridization is also shown. b) *In situ* hybridization using tumors transfected with Scr-shRNA#2. e) Quantitation of Ki67-positive cells. **Figure S11.** a) Immunofluorescence and neutral lipid staining, using tumor tissues transfected with Scr-shRNA#2. DAPI was used for counter staining of nuclei. Bar = 100 μm. b) Immunofluorescence using tumor tissues transfected with Scr-shRNA#2. Bar = 100 μm. Additional file 3: Table S2. Full list of genes (probes) identified by microarray analysis as shown in Additional file 2: Figure S4c. Additional file 4:
**Supplementary informations.** Reagents and experimental procedures [8,12,46].
