# Supplementary material for: Lipid starvation and hypoxia synergistically activate ICAM1 and multiple genes in an Sp1-dependent manner to promote the growth of ovarian cancer
Source: Mol Cancer. 2015 Apr 8;14:77. doi: 10.1186/s12943-015-0351-z (PMC4396815; doi:10.1186/s12943-015-0351-z)

Supplementary informations for Additional file 2

1) Figure S1a: The promoter regions of the *ICAM1*, *KLF6*, and *JUN* genes and the positions of Sp1, NFκB, and AP1 binding sites based on the current literature [19-21] are shown in the diagram. Black bars indicate PCR amplicon sizes for the ChIP assay. I: Input control lysate without immunoprecipitation. IgG: negative control using IgG. N and H indicate normoxia and hypoxia (1% O_2 for 16 h), respectively.
2) Figure S1, S2a,c, S3, S4, and S6: Cells were cultured for 16h.
3) Figure S2b, S5, S7a, and S8a: Cells were cultured for 24h.
4) Figure S2a,c, S3d,e, S4b, S5b,d, S6b, and S9a: Data shown are the mean (n = 3) ± SD.
5) Figure S1b: Data shown are the mean (n = 2) ± SD.
6) Figure S4a: α-tubulin and histone H3 were used as markers for cytoplasm and nuclei, respectively.
7) Figure S6a: Lanes were run on the same gel but were noncontiguous (separated by black line).
8) Figure S7a: Subsequently, cell blocks containing cells embedded in agarose and fixed in 10% neutral formaldehyde were prepared.
9) Figure S8b,c: Data shown are the mean (n = 3) ± SD. *P = 0.002; **P = 0.03.
10) Figure S9a: Cells were cultured under normoxia with FCS treatment, and then cell viability was determined by MTS assay.
11) Figure S9b, Tumors derived from OVISE cells transfected with shRNAs were isolated at day 27 post-cell injection.
12) Figure S10e: Data shown are the mean ± SD (N = 8). *P < 0.0001.

Figure S1

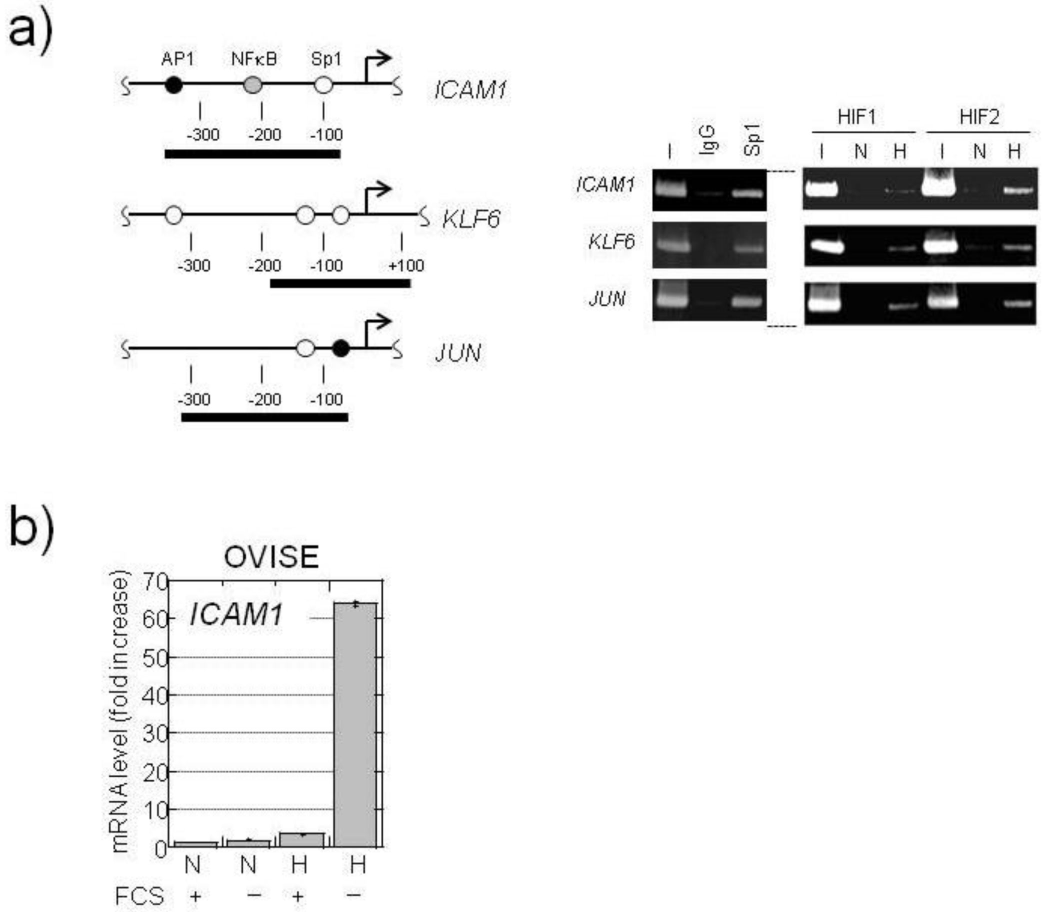
a)
AP1
NFκB
Sp1
ICAM1
-300
-200
-100
KLF6
-300
-200
-100
+100
JUN
-300
-200
-100
I
IgG
Sp1
HIF1
HIF2
I
N
H
I
N
H
ICAM1
KLF6
JUN
b)
OVISE
ICAM1
mRNA level (fold increase)
0
10
20
30
40
50
60
70
N
N
H
H
FCS
+
−
+
−

Figure S2

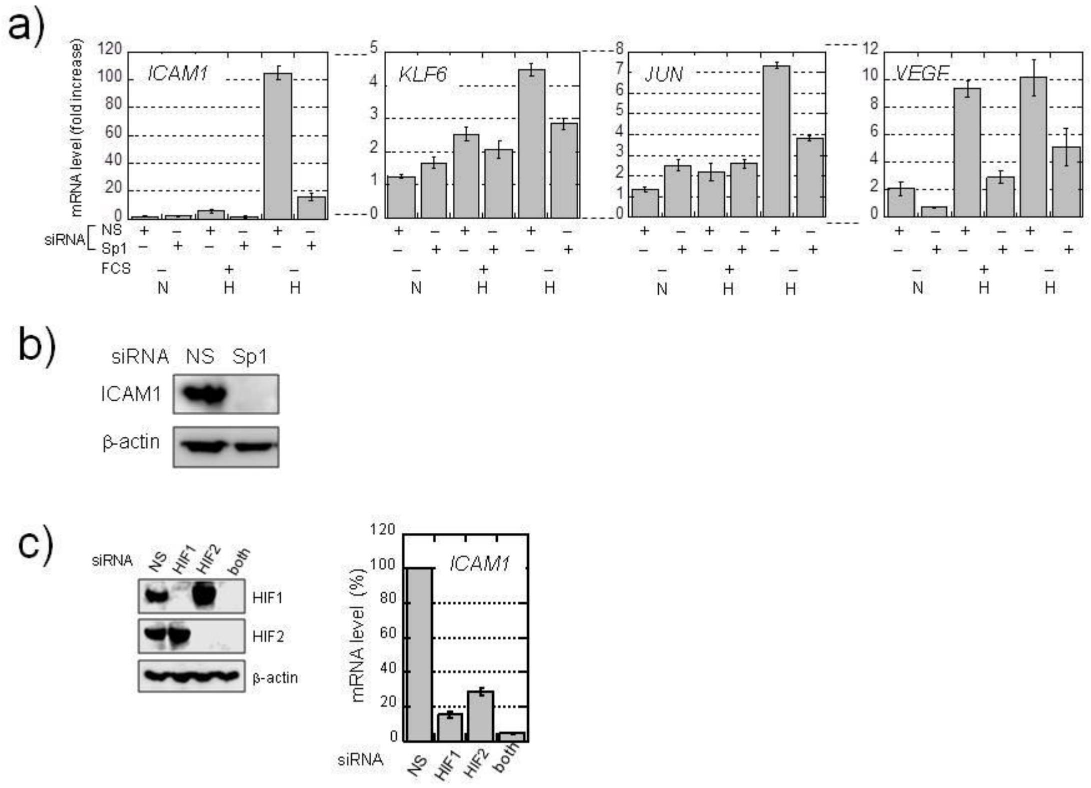
a)
mRNA level (fold increase)
ICAM1
KLF6
JUN
VEGF
siRNA
NS
Sp1
FCS
N
H
b)
siRNA NS Sp1
ICAM1
β-actin
c)
siRNA
NS
HIF1
HIF2
both
HIF1
HIF2
β-actin
mRNA level (%)
ICAM1

Figure S3

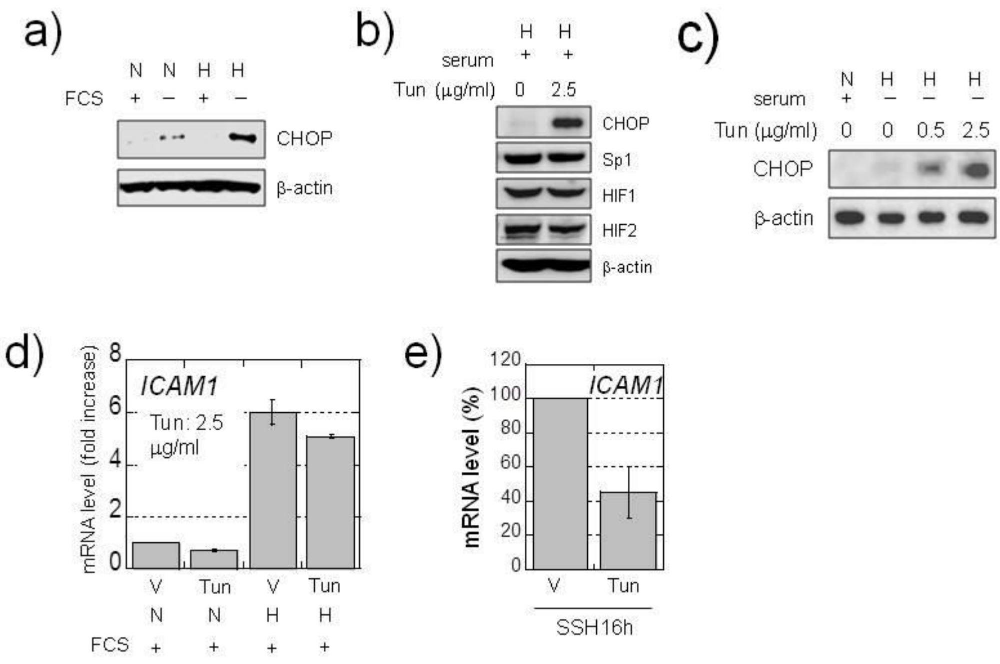

Figure S4

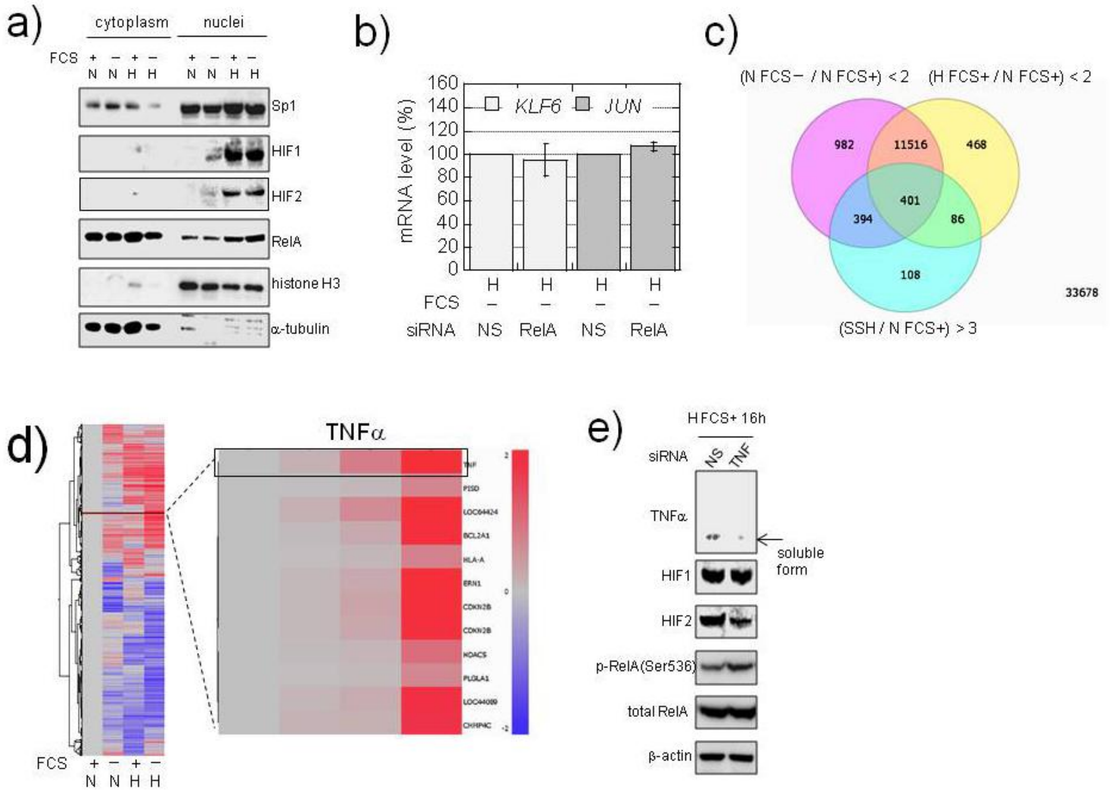

Figure S5

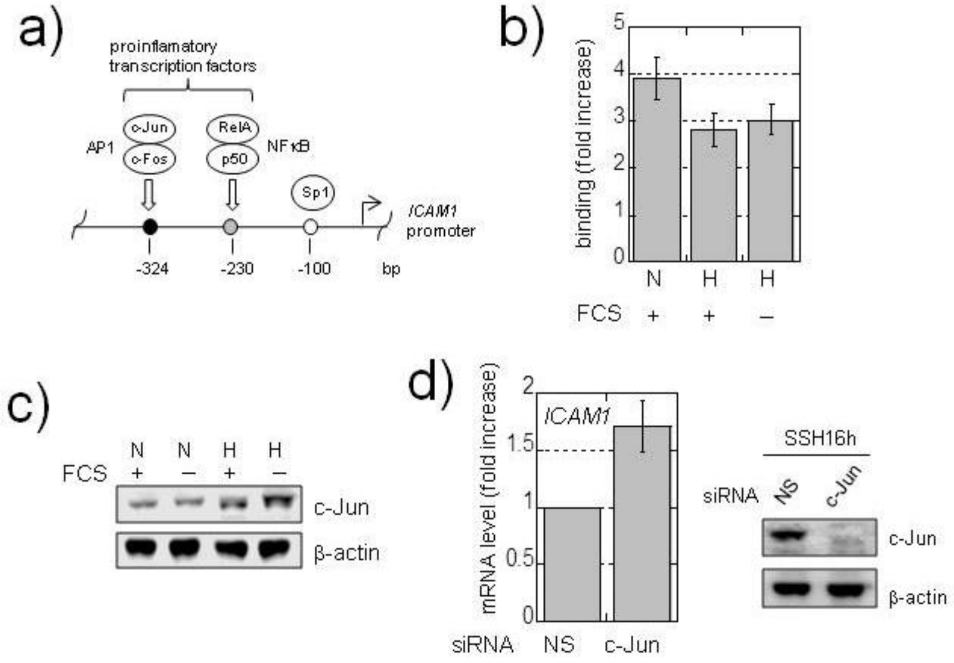

Figure S6

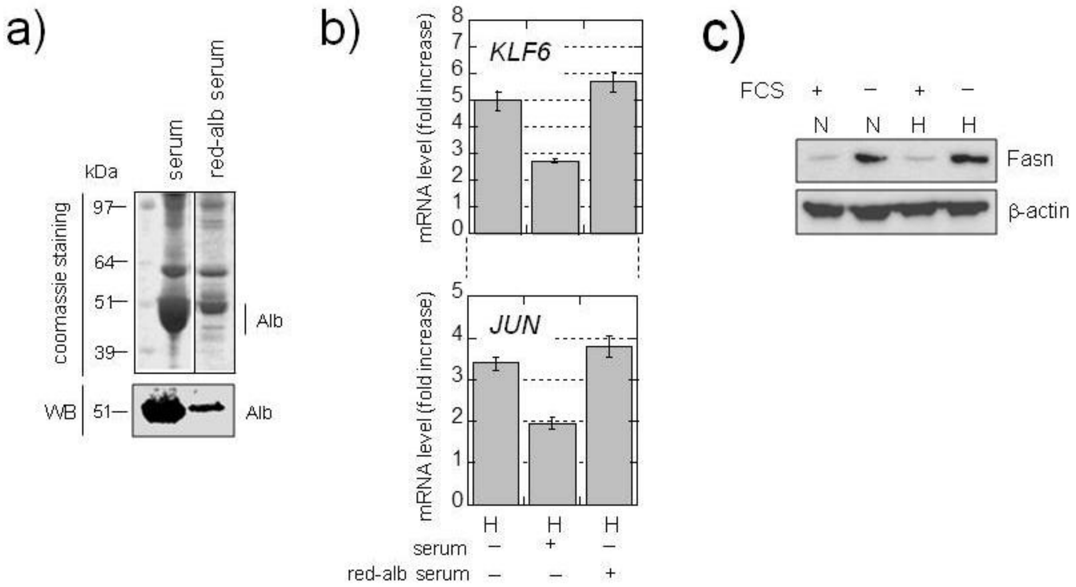

Figure S7

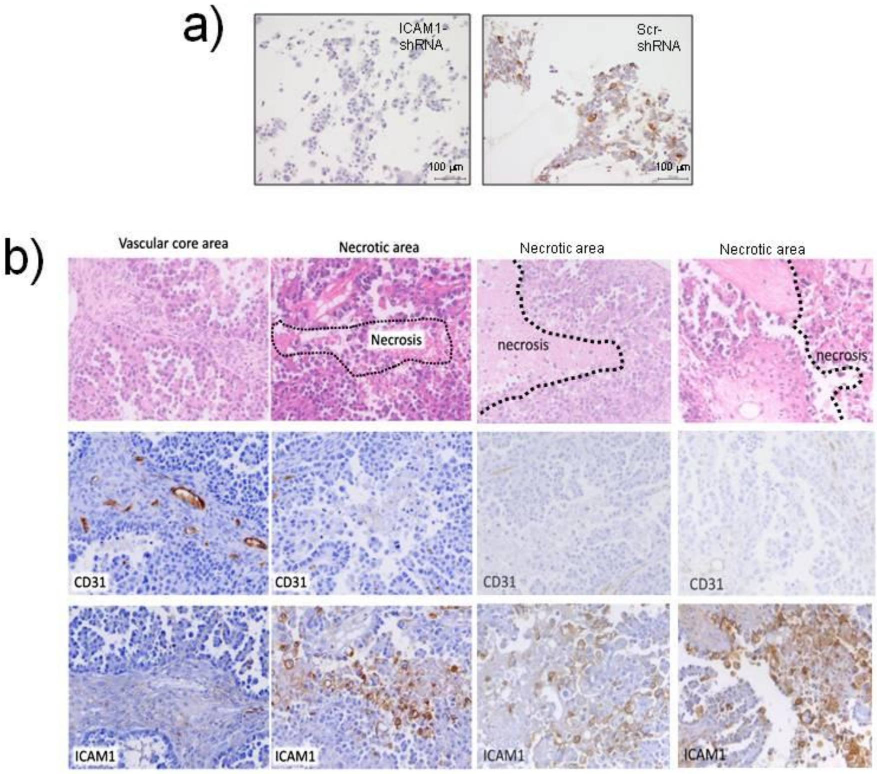
a)
ICAM1-
shRNA
100 µm
Scr-
shRNA
100 µm
b)
Vascular core area
Necrotic area
Necrotic area
Necrotic area
Necrosis
necrosis
necrosis
CD31
CD31
CD31
CD31
ICAM1
ICAM1
ICAM1
ICAM1

Figure S8

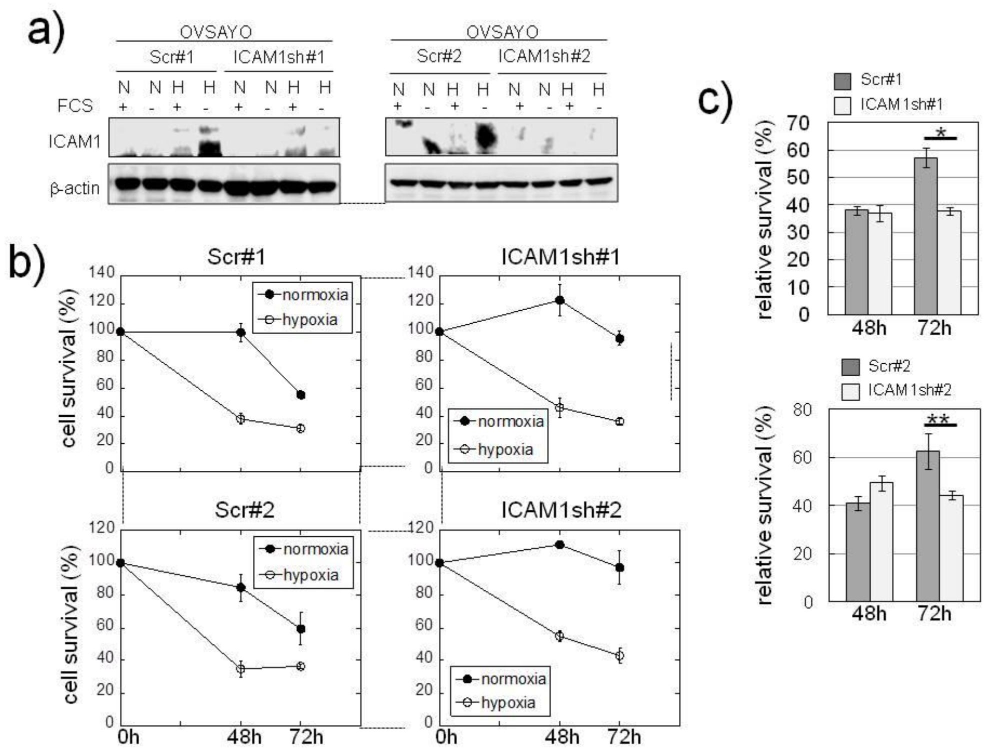

Figure S9

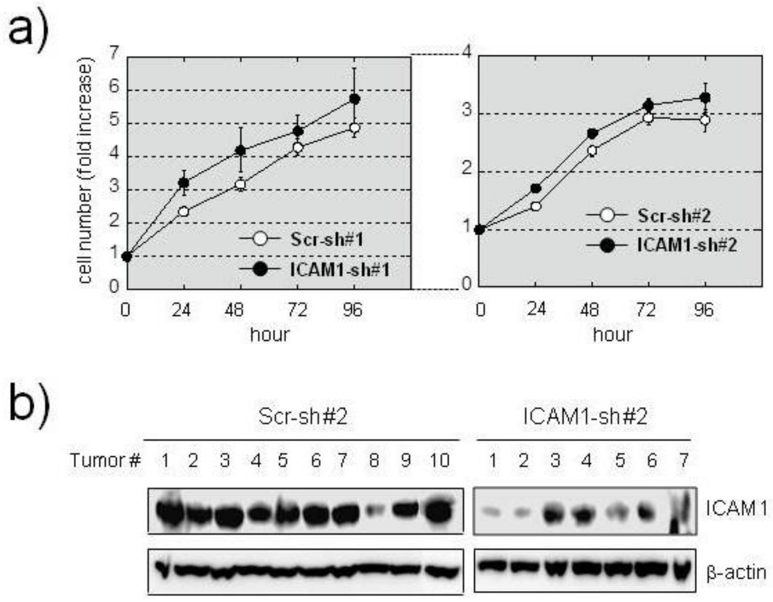

Figure S10

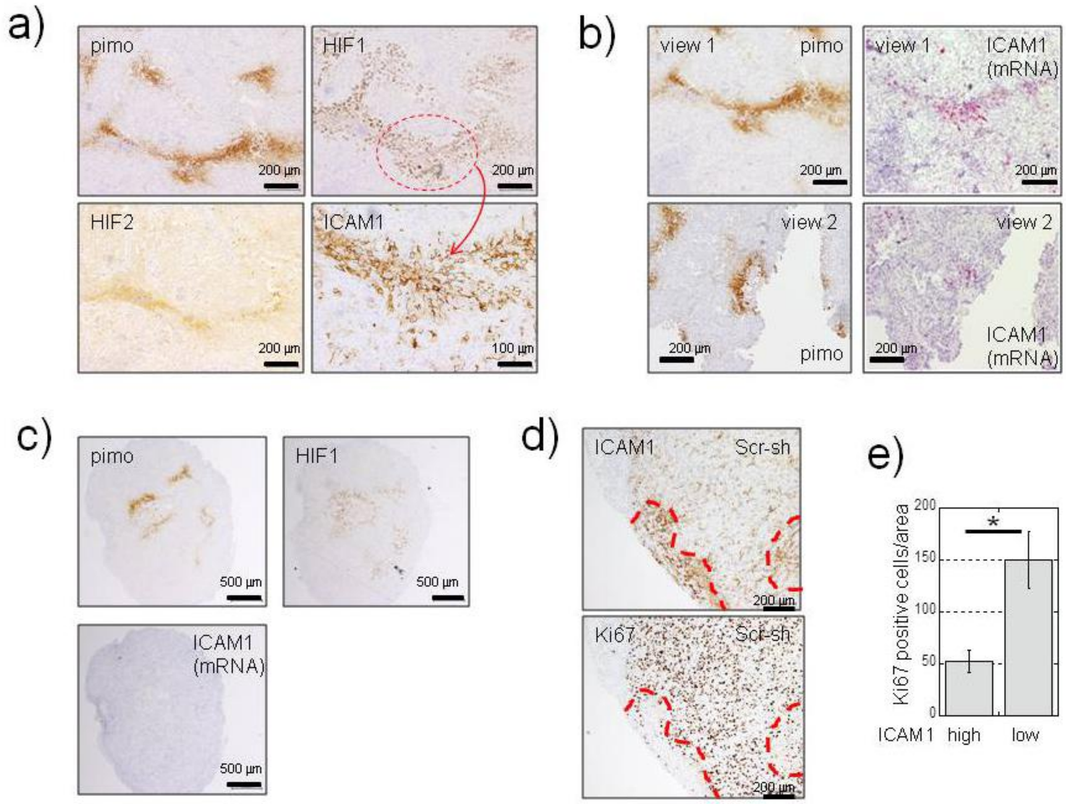

Figure S11

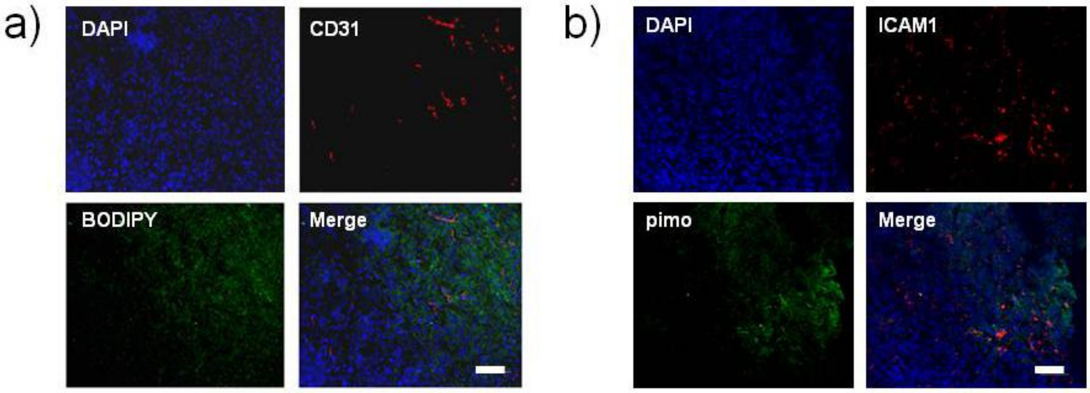

Supplement: Additional file 2: Figure S1. — a) ChIP analysis. b) ICAM1 expression in OVISE cells. Figure S2. a) Real-time RT-PCR analysis of genes in OVSAYO cells. b) Western blotting with OVSAYO cell lysates. c) Real-time RT-PCR analysis using cells cultured under SSH. Western blotting was also performed. Figure S3. a,b,c) Protein levels in OVSAYO cells. d) mRNA levels were analyzed for cells cultured with or without Tun. V: vehicle. e) Effect of Tun on the ICAM1expression. Figure S4. a) Western analysis of indicated fractions of OVSAYO cells. b) Effect of RelA on the activation of genes. c) Venn diagram representation of genes. d) Heat map representation of gene expressions. e) Western analysis of cells transfected with siRNA. Figure S5. a) AP1 and NFκB bind to the promoter region. b) Quantitative ChIP analysis of c-Jun binding to the ICAM1 promoter. c) c-Jun expression in OVSAYO cells. d) Real-time RT-PCR analysis. Cells transfected with siRNAs were cultured under SSH. c-Jun expression in siRNA transfected cells is also shown. Figure S6. a) Approximately 80% of albumin was removed from human serum, resulting in reduced-albumin serum. b) Real-time RT-PCR analysis of indicated genes. c) Expression of fatty acid synthase (Fasn) in OVSAYO cells. Figure S7. a) ICAM1 immunocytochemistry in OVISE cells transfected with Scr- and ICAM1-shRNA #1. Cells were cultured under SSH. b) H&E staining and IHC using a surgically removed specimens from CCC patients. Figure S8. a) Western blot analysis, b,c) MTS assay. Figure S9. a) Proliferation of OVISE cells transfected with shRNAs. b) ICAM1 expression in tumor tissues. Figure S10. a,c,d) IHC using tumors transfected with Scr-shRNA#2. In situ hybridization is also shown. b) In situ hybridization using tumors transfected with Scr-shRNA#2. e) Quantitation of Ki67-positive cells. Figure S11. a) Immunofluorescence and neutral lipid staining, using tumor tissues transfected with Scr-shRNA#2. DAPI was used for counter staining of nuclei. Bar = 100 μm. b) Immunofluorescence using t [file 12943_2015_351_MOESM2_ESM.pdf]
