# Supplementary material for: Lipid starvation and hypoxia synergistically activate ICAM1 and multiple genes in an Sp1-dependent manner to promote the growth of ovarian cancer
Source: Mol Cancer. 2015 Apr 8;14:77. doi: 10.1186/s12943-015-0351-z (PMC4396815; doi:10.1186/s12943-015-0351-z)
Supplement: Additional file 4: — Supplementary informations. Reagents and experimental procedures [8,12,46]. [file 12943_2015_351_MOESM4_ESM.docx]

**Supplementary informations**

siRNA

ON-TARGET plus SMART pool reagents (Dharmacon, Lafayette, CA, USA) were used for siRNAs against ARNT, mTOR, and TNFα. siRNAs targeting RelA (h, sc-29410), c-Jun (h2, sc-44201), Sp1 (h2, sc-44221), HIF1α (h2, sc-44225), EPAS-1 (h, sc-35316), and mTOR mRNA (h, sc-35409) were purchased from Santa Cruz Biotechnology, Santa Cruz, CA, USA. In additional, Silencer Select siRNAs against RelA (s11915) and TNFα (s14248) were purchased from Life Technologies. Ambion Silencer Negative Control #1 siRNA (Life Technologies) and an additional non-specific siRNA previously described [46] were used as non-specific siRNAs. Transfection of siRNAs was performed using Lipofectamine RNAi MAX (Life Technologies). HIF-specific siRNAs were used at a final concentration of 20 nM and all other siRNAs were used at 40 nM.

Antibodies

Antibodies used for western blots against Sp1, HIFs, ARNT, β-actin, and CHOP were described previously [12]. Antibodies against mTOR (2972), phosphor-mTOR (2971), phospho-RelA (3033), TNFα (6954), and phospho-c-Jun (9261) were obtained from Cell Signaling Technology (Danvers, MA, USA) and used at a 1:2500 dilution. Antibodies against ICAM1 (sc-8439, 300 ng/ml), RelA (p65; sc-372, 200 ng/ml), and FASN (sc-55580, 300 ng/ml) were from Santa Cruz. Additional antibodies: c-Jun (ab31419, Abcam, 300 ng/ml), albumin (R1048P, Acris Antibodies GmbH, Herford, Germany, 300 ng/ml), histone H3 (ab1791, Abcam, 200 ng/ml), PARP-1 (E78, Epitomics, Burlingame, CA, USA, 1/3000 dilution), and α-tubulin (T-9026, Sigma, St. Louis, MO, 1/2500 dilution).

Real-time RT-PCR analysis

The sequences of primers and probes used in this study are shown below.

| Gene | Primer sequences | Probe sequences |
| --- | --- | --- |
| *ICAM1* | 5′-CAGAGGTTGAACCCCACAGT-3′ (forward)  5′-CCTCTGGCTTCGTCAGAATC-3′ (reverse) | 5′-CAGAGCCAGGAGACACTGCAGACAGTG-FITC-3′ and 5′-LCRed640-CATCTACAGCTTTCCGGCGCCCA-p-3′ |
| *KLF6* | 5′-CCATGTGCAGCATCTTCCAGG-3′ (forward)  5′-GGCTCGCTCTGGAGGTAACG-3′ (reverse) | 5′-GACCGGCTACTTCTCGGCGCTGC-FITC-3′  and 5′-LCRed640-GTCTCTGGAGGAGTACTGGCAACAGAC-p-3′ |
| *JUN* | 5′-GCTGAGGTTTGCGTAGACCGG-3′ (forward)  5′-TCAACGGGGCAGGCATG-3′ (reverse) | 5′-GGTAGCCTCGGTGGCAGGGGG-FITC-3′  and  5′-LCRed640-AGCGGCAGCGGCGGCTTC-p-3′ |
| *AR* | 5′-TCAGTTCACTTTTGACCTGCTAA-3′ (forward)  5′-GTGGAAATAGATGGGCTTGA-3′ (reverse) | 5′-CACATGGTGAGCGTGGACTTTC-FITC-3′  and  5′-LCRed640-GAAATGATGGCAGAGATCATCTCTGT-p-3′ |

ChIP assay

The sequences of primers used in this study are shown below. Five micrograms of antibodies were used for immunoprecipitation in each ChIP experiment. PCR was performed using ExTaq-HS (Takara, Shiga, Japan) for amplification of *ICAM1*, and *JUN.* LA-Taq (Takara) was used to amplify *KLF6*. Cycle conditions were 95°C for 2 min, followed by 35 cycles of 95°C for 30 sec, 56°C for 20 sec, and 72°C for 30 sec for *KLF6* and *JUN*, or 35 cycles of 95°C for 30 sec, 58°C for 20 sec, and 72°C for 30 sec for *ICAM1*. Antibodies against RelA (sc-372) and c-Jun (ab31419) were purchased from Santa Cruz Biotechnology and Abcam, respectively. The other antibodies used in this study have been described elsewhere [8,12].

| Gene | Primer seqeunces |
| --- | --- |
| *ICAM1* | 5′-GTGTAGACCGTGATTCAAGCTTAGC-3′ (forward)  5′-GCGCGTGATCCTTTATAGCGC-3′ (reverse) |
| *KLF6* | 5′-TGGCCGGAGCTGACATCATC-3′ (forward) 5′-CCGAAAGTCTTCCCGGAGC-3′ (reverse) |
| *JUN* | 5′-AGGGGACCGGGGAACAGAG-3′ (forward) 5′-CCCCTAAAAATAGCCCATGATGTC-3′ (reverse) |

Primers and probes used for quantitative ChIP analysis were as follows.

| gene | primers | probes |
| --- | --- | --- |
| *ICAM1* | 5′-CGATTGCTTTAGCTTGGAAATTCC-3′ (forward) 5′-GCGCGTGATCCTTTATAGCGC-3′ (reverse) | 5′-CCCTGTCAGTCCGGAAATAACTGC-FITC-3′ and 5′-LCRed640-GCATTTGTTCCGGAGGGGAAGGC-p-3′ |

Reporter gene assay

All PCR fragments prepared from genomic DNA of OVSAYO cells were inserted into the luciferase vector at the *Kpn*I and *Xho*I sites. Mutant constructs were prepared using the QuikChange Mutagenesis Kit (Stratagene, La Jolla, CA, USA). Promoter fragments of *ICAM1* (positions 1531–1808, Accession No. AY225514), *KLF6* (positions 1312–1627, Accession No. AF284036), and *JUN* (positions 8–240, Accession No. J04111) were amplified by PCR and inserted into the pGL4.10 plasmid (Promega) at the *Kpn*I/*Xho*I sites. Promoter fragments of *ICAM1* and *KLF6* were amplified using KOD-FX DNA Polymerase (TOYOBO, Tokyo, Japan). A *JUN* promoter fragment was amplified using KOD-plus DNA Polymerase (TOYOBO) and then analyzed by nucleotide sequencing. The sequences of primers used in this study are shown below.

| Constructs | Primers |
| --- | --- |
| *ICAM1* promoter | 5′-GGGGTACCGTGTAGACCGTGATTCAAGCTTAGC-3′ (forward) 5′-CCGCTCGAGGCGCGTGATCCTTTATAGCGC-3′ (reverse) |
| *KLF6* promoter | 5′-GGGGTACCTGGCCGGAGCTGACATCATC-3′ (forward)  5′-CCGCTCGAGCCGAAAGTCTTCCCGGAGC-3′ (reverse) |
| *JUN* promoter | 5′-GGGGTACCAGGGGACCGGGGAACAGAG-3′ (forward) 5′-CCGCTCGAGCCCCTAAAAATAGCCCATGATGTC-3′ (reverse) |
| Sp1 site mutant 1 | 5′-GGGAAACGGGAGAGCAGCCCCCGG-3′ (forward) 5′-CCGGGGGCTGCTCTCCCGTTTCCC-3′ (reverse) |
| Sp1 site mutant 2 | 5′-GGAAAGCAGCACTTGGCCCCCAG-3′ (forward) 5′-CTGGGGGCCAAGTGCTGCTTTCC-3′ (reverse) |

Small hairpin RNAs

The sequences of shRNAs used (without 5′ overhangs) are as follows.

| shRNA | Sequences |
| --- | --- |
| Scramble | 5′-GAAATGTACTGCGCGTGGAGACGTTTTGGCCACTGACTGACGTCTCCACGCAGTACATTT-3′ |
| ICAM1#1 | 5′-TAAGGTTCTTGCCCACTGGCTGTTTTGGCCACTGACTGACAGCCAGTGCAAGAACCTTA-3′ |
| ICAM1#2 | 5′-GTTGAATAGCACATTGGTTGGCGTTTTGGCCACTGACTGACGCCAACCAGTGCTATTCAA-3′ |
